# A Vendor-Independent Mobile Health Monitoring Platform for Digital Health Studies: Development and Usability Study

**DOI:** 10.2196/12586

**Published:** 2019-10-29

**Authors:** Thijs Vandenberk, Valerie Storms, Dorien Lanssens, Hélène De Cannière, Christophe JP Smeets, Inge M Thijs, Tooba Batool, Yves Vanrompay, Pieter M Vandervoort, Lars Grieten

**Affiliations:** 1 Faculty of Medicine and Life Sciences Hasselt University Diepenbeek Belgium; 2 Future Health Department Hospital Oost-Limburg Genk Belgium; 3 Department of Obstetrics & Gynecology Ziekenhuis Oost-Limburg Genk Belgium; 4 Department of Cardiology Hospital Oost-Limburg Genk Belgium; 5 Transportation Research Institute (IMOB) Hasselt University Hasselt Belgium

**Keywords:** information science, patient care management, mobile health, telemonitoring, monitoring, ambulatory

## Abstract

**Background:**

Medical smartphone apps and mobile health devices are rapidly entering mainstream use because of the rising number of smartphone users. Consequently, a large amount of consumer-generated data is being collected. Technological advances in innovative sensory systems have enabled data connectivity and aggregation to become cornerstones in developing workable solutions for remote monitoring systems in clinical practice. However, few systems are currently available to handle such data, especially for clinical use.

**Objective:**

The aim of this study was to develop and implement the digital health research platform for mobile health (DHARMA) that combines data saved in different formats from a variety of sources into a single integrated digital platform suitable for mobile remote monitoring studies.

**Methods:**

DHARMA comprises a smartphone app, a Web-based platform, and custom middleware and has been developed to collect, store, process, and visualize data from different vendor-specific sensors. The middleware is a component-based system with independent building blocks for user authentication, study and patient administration, data handling, questionnaire management, patient files, and reporting.

**Results:**

A prototype version of the research platform has been tested and deployed in multiple clinical studies. In this study, we used the platform for the follow-up of pregnant women at risk of developing pre-eclampsia. The patients’ blood pressure, weight, and activity were semi-automatically captured at home using different devices. DHARMA automatically collected and stored data from each source and enabled data processing for the end users in terms of study-specific parameters, thresholds, and visualization.

**Conclusions:**

The increasing use of mobile health apps and connected medical devices is leading to a large amount of data for collection. There has been limited investment in handling and aggregating data from different sources for use in academic and clinical research focusing on remote monitoring studies. In this study, we created a modular mobile health research platform to collect and integrate data from a variety of third-party devices in several patient populations. The functionality of the platform was demonstrated in a real-life setting among women with high-risk pregnancies.

## Introduction

### Background

The increasing penetration of smartphones into consumer markets, as well as the growth in connected devices for health care, sport, and wellness, is leading to a dramatic increase in consumer-generated data [1]. Smartphones are becoming increasingly integrated into the global population. The number of smartphone users increased by nearly 1 billion people between 2014 (1.57 billion) and 2017 (2.32 billion), with an expected further increase to 2.87 billion users by 2020 [2].There are currently more than 259,000 health-related smartphone apps available in Web-based app stores; most of these apps were developed for Android (Google, CA, USA)and iOS (Apple Inc, CA, USA) [3]. The same technologies and concepts can also be applied to support health care. Remote monitoring of patients’ vital signs and behaviors in their home environment could play an essential role in achieving a sustainable and high-quality health care system [4,5] and reduce health care costs [3,5,6] by offering near-continuous patient follow-up, better data management strategies, and better treatment adherence. Some examples of chronic diseases that could benefit from this type of platform include diabetes, heart failure, and cardiac arrhythmias [7-9]. The information obtained by remote monitoring provides an additional dimension to that possible with standard clinical care that is undertaken mainly by spot checks in the clinic. Continuous real-time patient tracking and processing of various parameters will influence the way health care practitioners deal with prevention, diagnostics, and disease management.

It is already possible to connect mobile health apps to a wide range of portable devices or sensors [10], such as those used to measure physiological parameters (eg, blood pressure, blood glucose, weight, and activity) [11]. An electrocardiogram (ECG) can be recorded via an app connected to a wearable ECG device [12]. In addition to the advantages for patients, doctors will also benefit from mobile health tools. Mobile apps can be used as an interface between the patient and the clinician via a communication channel [10,13], which could act as a substitute for some ambulatory visits [14]. As they enable patients to be monitored in their home environment, mobile health tools could prevent some hospitalizations or shorten the hospital stay [3,6] and have positive effects on medical outcomes, health care expenditure [6], and quality of life. These benefits would be due partly because the patient stays in their familiar environment and partly because continuous monitoring gives the opportunity for immediate intervention, if needed [10].

### Digital Equipment

Remote monitoring patients at home requires a data communication system between the patient and the health care professional. Typically, the patient is equipped at home with a set of sensors that measure vital signs and a smartphone app that collects data on behavior, symptoms, and location. These data are collected on a cloud-based server that is accessed by the health care professional. Tools for decision support to assist the health care professional and semiautomatic feedback to patients are necessary to manage large patient populations.

Despite the significant technological advances in developing novel sensory systems and state-of-the-art devices, there has been limited investment in developing the infrastructure that is required to connect and handle the amount of information that these devices generate. In particular, there are limited tools available to handle this health information in terms of clinical applications [15]. With the rapid increase in novel tools and technologies, data connectivity and aggregation have become the cornerstones in developing workable solutions to manage patients in clinical practice and support scientific research in the provision of digital health [16]. However, the commercial remote monitoring technology market is highly fragmented because each vendor has developed their own data platform to record data from their associated sensors and communicate these using stand-alone software solutions or Web-based apps. Accordingly, it is impossible to aggregate data obtained from the sensors developed by different vendors. Clinical practice and academic research that rely on remote monitoring are limited by these closed, manufacturer-owned platforms. Therefore, a generic and open digital research platform for remote monitoring is needed to allow academic and clinical research into remote monitoring. The platform is required to overcome the problems of third-party device integration and the collection of various data feeds from patient populations.

### Objectives

In this paper, we discuss the development of a generic and open digital research platform for remote monitoring that can be used to perform academic and clinical research into remote monitoring. This digital platform overcomes the problems of third-party device integration and collection of various data feeds from patient populations. The collected data can be analyzed using the platform and presented visually to the caregiver. We demonstrate the functionality of the platform in a real-life setting, among women with high-risk pregnancies.

## Methods

### Overview

The concept and design of our modular digital health research platform for mobile health (DHARMA) platform were focused primarily on clinical usability. Accordingly, health care professionals (doctors and nurses) participated in each stage of its conceptualization and development, from design and prototyping through to implementation and user trials.

The DHARMA platform was developed in close collaboration with all stakeholders: doctors, professional caregivers, researchers, and experts in mobile health. As its primary aim is to support clinical studies, the platform was built taking into account the requirements of specific studies but always with general applicability and genericity in mind. We ensured short test cycles by using the agile development methodology (Scrum). An appropriate toolset was put into place for managing user stories (Jira), documenting design and implementation decisions (Confluence), and continuous build and deployment (Gitlab). To modularize implementation, the GitFlow approach was used. To separate continuous building and release versions, deployment was split up into a development, test, and production environment. Teams collaborated efficiently using Slack. For components of specific studies, the core development team was extended by internship students.

The app comprises a Web-based user interface and an object-oriented programming language (Java)–hypertext preprocessor (PHP) back end. The platform is set up on a cloud server. The front and back ends are coded in various languages. The front end is built with PHP with a combination of bootstrap and Laravel frameworks. The back end is built with a combination of PHP and Java to ensure cross-platform compatibility. The server runs on Windows, Apache, MySQL, and PHP platforms. Owing to the highly valuable content of the database system, the database is backed up daily and archived.

### Technical Architecture and Data Security

The platform was built as a pluggable component-based middleware. As mentioned by Piwek et al [17], data security and patient privacy are essential to the adoption of digital smartphone research methods. A centralized data structure and shared research platform for multiple studies, as proposed in this study, eliminates the need to develop individual data security solutions for individual studies [18]. Given the use of highly sensitive data, the Health Insurance Portability and Accountability Act, especially the technical regulations, was used to achieve the highest possible level of security. We also followed the privacy by design principle of the general data protection regulation (GDPR). Strict security protocols are in place to ensure data safety, including several firewalls and secure sockets layer certificates embedded in the cloud-hosting infrastructure, virtual server, and database. OpenVPN is implemented to ensure a safe connection between the personal computer (PC) and the server. The database is encrypted by cipher block chaining in combination with advanced encryption standard (AES). User authentication for access to the platform is handled by a user login protected by Google’s reCAPTCHA technique. All false login attempts are logged in the database, revealing unwanted access attempts and allowing us to create internet protocol exclusion rules. Communication between the database and the platform is encrypted by the AES-Rijndael algorithm. All data exchange with external databases is performed using the handshake principle, based on a standardized OAuth verification/authentication procedure.

### Components

The concept of components was introduced to manage different studies on a single platform. Each study comprises at least 1 or multiple components (eg, data handling and questionnaires) that can be activated by a study leader.

#### Study and Patient Administration

Every clinical study is divided into multiple levels, starting with the hospital acting as the lead partner of the clinical study (level 1; eg, Hospital East-Limburg, Genk, Belgium). The next level is the medical domain (level 2; eg, gynecology), and the third level is the study itself (level 3; eg, pregnancy remote monitoring study, Premom). This multilevel approach allows us to implement user rights and develop alert thresholds on each level of the study. Currently, 3 user profiles are defined: patient, study leader, and administrator (admin). After completing the registration process, 2 possible methods are provided. First, the admin can appoint a user as a study leader, and then a study leader can assign the user as a patient for inclusion in a specific study. This hierarchical model allows study leaders to independently create and follow-up patients.

#### Data Handling

**Figure 1:**
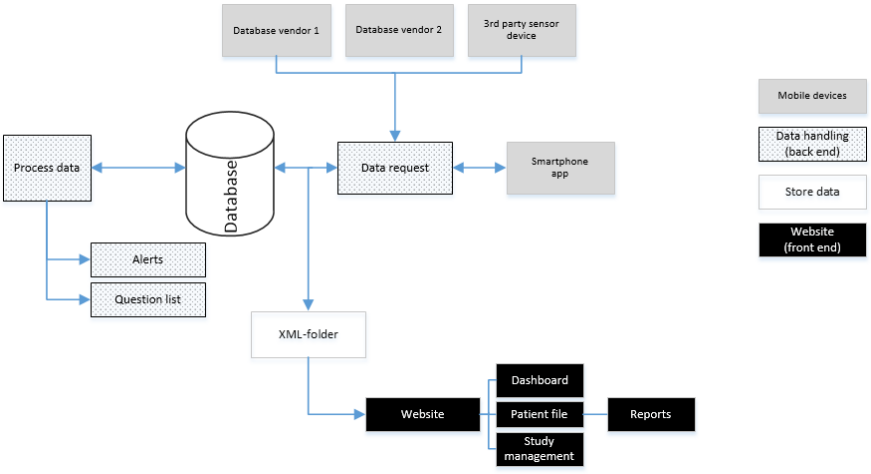
Overview of the data flow of the digital health research platform for mobile health platform.

The goal of DHARMA is to aggregate and visualize multiple vital parameters collected by using devices and medical apps from different vendors without the need to consult the vendor-specific platforms. The remote monitoring platform can receive information directly or collect data by connecting to other databases. If new values are uploaded by a user, DHARMA receives a notification and automatically launches the technical process needed to aggregate and store the data in its own database. Duplication of these data enables secure storage and accessibility for analysis and alert generation. Figure 1 is a graphic representation of the data flow in DHARMA. As the sensors record diverse types of data, ranging from discrete values to longitudinal values collected throughout the day, a metamodel was designed to handle and store data from each sensor. To handle large amounts of data, such as intraday (minute-by-minute) results from an activity tracker, data are compressed into a tailored XML file that is stored in a folder structure on the server.

To generate an efficient data collection workflow, an alert engine was developed, which can interpret and handle medical and technical alerts. Medical alerts are based on the collected data and detect a value outside the specified thresholds. The thresholds are specified for each study (level 3) and can dynamically support longitudinal changes and more complex interpretations. However, the configuration of patient-level alerts can be set to individual ranges based on clinical guidelines. On the basis of the clinical input, the alerts are categorized as normal, medium, or high priority. Technical alerts are defined by messages containing information about missed data transmissions. This triage system could help organize the clinical call center activities.

#### Question Management

The *question management* component was implemented to receive additional, context-related information from patients via questionnaires. Context information could help researchers interpret (different) vital parameters [19]. Web- and app-based questionnaires can be developed and linked to DHARMA. For Web-based questionnaires, the URL for the questionnaire is sent to the patient’s email address, and the patient can log in to the platform to answer the questionnaire. We developed the DHARMA smartphone app for app-based questionnaires. This app was built with the cross-platform Xamarin language to enable a solution compatible with both Android and iOS. The user interface was developed with Xamarin.Forms. A secured oauth2 app programming interface (API) connection between the platform and the app was developed to handle data flow. A local smartphone SQLite database is used to save and answer questionnaires offline. The app was integrated with firebase cloud messaging to notify patients when a questionnaire is delivered to the smartphone app. Questionnaires can be sent automatically via email or via the smartphone app by the Laravel task scheduler. This script comprises the questionnaire identifier (ID), study name, and date and time stamps. Questionnaires can be developed by the study leaders or admins and comprise individual questions. Each question has an identical ID and can be used in multiple questionnaires. Questions can be written as open questions, multiple choice questions, yes/no questions, or scale.

#### Patient File

A patient file component was created to arrange the information into individual patient records. Each record comprises 4 main tabs: medical information, statistics, questionnaires, and follow-up. The medical information tab allows the study leader and the clinician to view study-specific patient parameters or comorbidities. Study parameters include the study-specific information needed to interpret the vital parameters. The statistics tab displays a graphical overview of each vital parameter. The questions tab provides an overview of the questionnaires that were sent to and completed by the patient. The follow-up tab allows caregivers to send text messages among multiple disciplines. Each patient contact is logged in this tab.

#### Reports

A report component was created to provide a comprehensive digital overview of the patient’s status. The overview can be printed or downloaded and emailed to the patient’s doctor or caregiver. Lava Charts (Google Chart API) was used to visualize the patient’s data in charts and graphs.

## Results

The remote monitoring study platform was built between February 2015 and July 2018. The custom-made remote monitoring platform was deployed to monitor patients outside the hospital in several studies, including multiple sclerosis, low back pain, and osteoporosis studies in which the patients’ activity data (number of steps and intensity) were tracked.

### Overview of Platform Data

Table 1 provides an overview of the main studies in which DHARMA was tested, together with the numbers of patients and the vital parameters. Each vital parameter displays the number of individual measurements that were uploaded during the study.

**Table 1:** Remote studies included in digital health research platform for mobile health.

Study	Sample size, n	Blood pressure, n	Weight, n	Activity data, n
Premom	604	95,835	9430	35,520
Multiple sclerosis	36	0	14	1544
Low back pain	33	12	110	2191
Osteoporosis	13	25	857	1603

The main study in which DHARMA was tested was the Premom [20-22]. Briefly, this prospective cohort study enrolled pregnant women at high risk of developing pre-eclampsia. Patients were provided with a commercial activity tracker, wireless blood-pressure monitor, and a smart body scale analyzer from iHealth (iHealth Labs Inc, Mountain View, CA, USA) or Withings (Withings, Issy-les-Moulineux, France). The participating women were asked to measure their blood pressure twice daily, their weight once daily, and wear an activity tracker for 24 hours per day. All of the information recorded by the devices was sent wirelessly to the smartphone, which then transmitted the data to the Web-based platform for aggregation. The sensors collected up to 12 different signals as functions of time, enabling multiparametric longitudinal research. A midwife reviewed all incoming remote monitoring data via the dashboard. The alert engine discriminated between normal and alarm signals for the following: systolic blood pressure >140 mmHg, diastolic blood pressure >90 mmHg, or weight gain >1 kg per day. Alarm events were sent to the obstetrician to discuss possible interventions.

### Dashboard and Visualization

**Figure 2:**
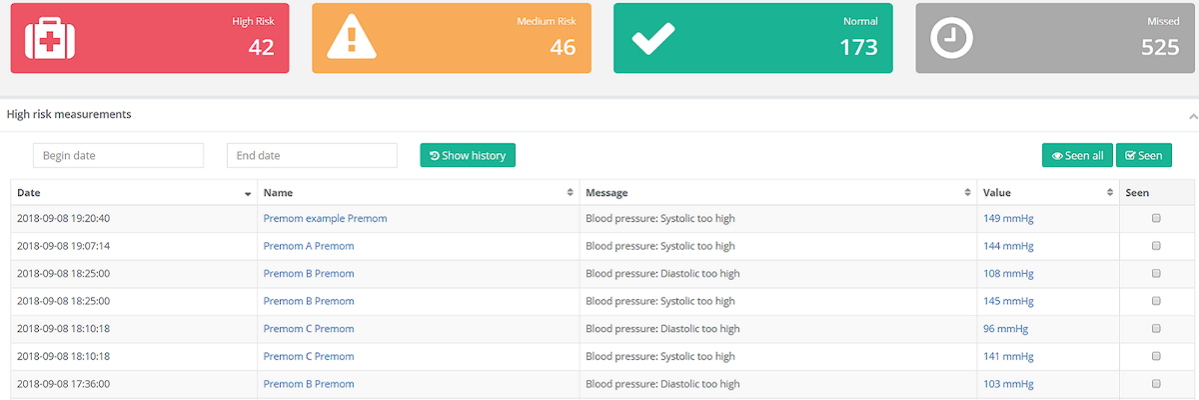
Screenshot of the alert representation upon login of the dashboard. Data was triaged based on predetermined thresholds into High Risk, Medium Risk or Normal.

Besides collecting and handling different sources of information, one of the main objectives of the platform is to provide the researcher/clinician with efficient visualization of all patient data. If the data triggered a specific alert, the dashboard prioritized the alerts based on the predefined thresholds and displayed them to the person responsible for reviewing the data. This enables the platform to triage patient alerts and facilitate patient handling and follow-up. Figure 2 shows an overview of the dashboard.

### Patient File

**Figure 3:**
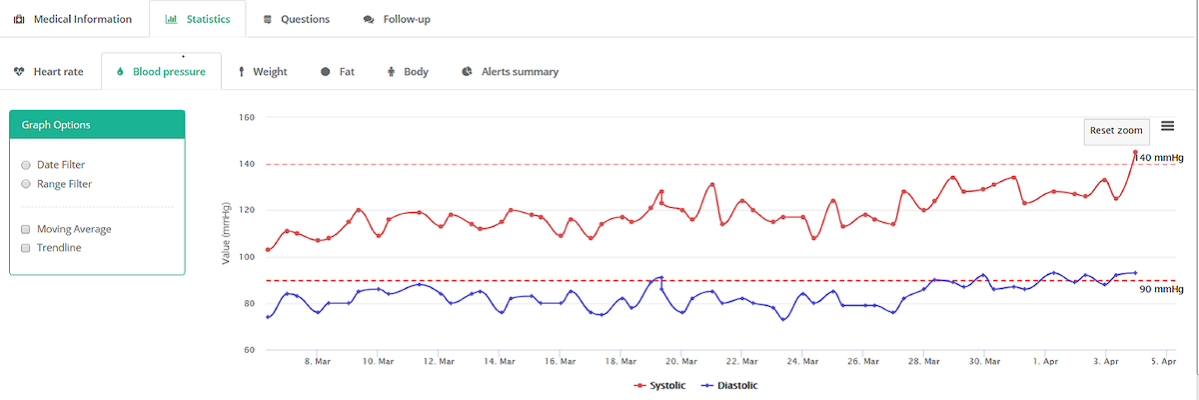
Overview graph of a patient’s blood pressure displaying both the systolic and diastolic blood pressure, with the predefined alert thresholds indicated with dashed lines.

The patient records bundle the individual patient’s information into a single file. The received parameters are individually plotted as functions of time to identify specific trends that could trigger an alert by crossing specified thresholds. For the patient shown in Figure 3, systolic blood pressure showed a trend toward crossing the predefined thresholds (140 mmHg for systolic blood pressure and 90 mmHg for diastolic blood pressure), triggering a high-risk alert. On the basis of these results, the patient was admitted to hospital where early symptoms of pre-eclampsia were identified, and appropriate treatment was started.

### Data Structure and Data Handling: Activity Data

**Figure 4:**
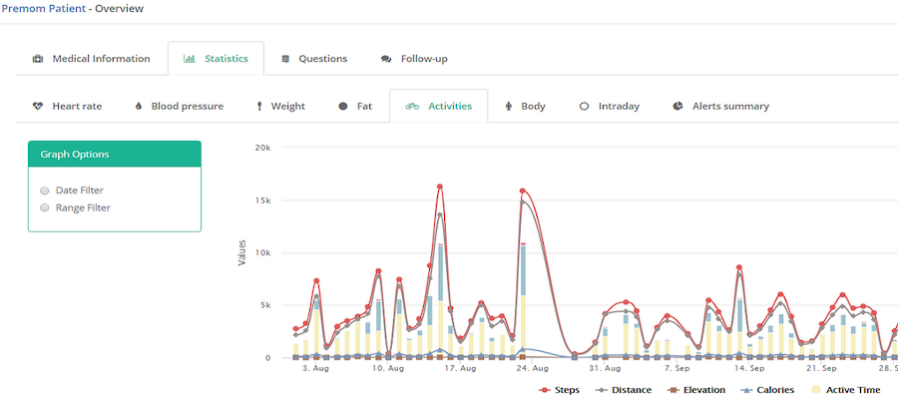
An example of longitudinal activity data for a period of about 2 months.

**Figure 5:**
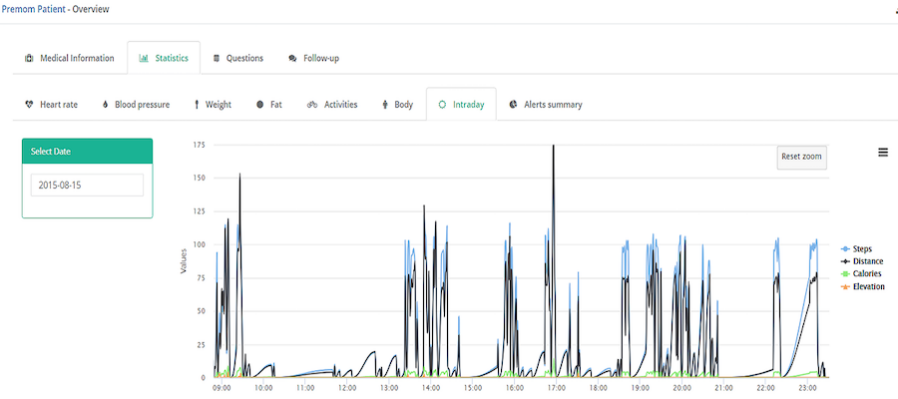
Granular example of the activity level per minute for a period of about 15 hours.

The challenges of working with different types of data include how to handle, analyze, and store the data appropriately and transparently. In the normal workflow, only the summarized values of larger datasets are used because granular detail is not required for daily patient management. However, if required, the data are available for Web-based data processing or can be exported for offline scientific research, such as the development of novel algorithms or data-processing techniques. Figure 4 shows the average step count over a period of 12 weeks. Granular, minute-by-minute data are also shown for a single day in Figure 5.

## Discussion

### Principal Findings

This paper outlines the development of a digital health research platform for remote monitoring. By combining advanced wearable sensors with smartphone technologies for remote monitoring, it is possible to monitor the health of patients in their home environment, an approach that may reduce the number of *health care visits.* Remote monitoring requires multiple hardware and infrastructure tools. Each vendor provides dedicated infrastructure and data review platforms specific to their own devices. Accordingly, data aggregation is impossible when collating data from medical devices and tools from different manufacturers, creating a barrier to clinical practice and academic research. This fragmentation is also very inconvenient for the user. Therefore, a *one size fits all* solution (ie, one platform for all devices) is highly desirable [23].

A health monitoring system can only provide its greatest usability if it can be fully integrated into the user’s and the physician’s daily workflow. The goal of our study is to integrate the data streams from multiple medical device vendors and allow health care practitioners to efficiently evaluate a patient’s health status. This will improve efficiencies in cost and time. In addition, the platform was designed to enable rapid and cost-effective scalability.

Privacy is a fundamental right in the public health care domain, especially following the recently implemented GDPRs. Health care practitioners and patients are becoming increasingly aware of this important aspect. Confidential handling and storage of private patient data have also become a critical aspect of study design. Therefore, all personal data in our platform are deidentified and every unique identification number, characteristic, or name is removed. Moreover, all participants need to provide signed written informed consent.

### Comparison With Previous Studies

The development of a centralized visualization platform has been described in earlier reports, for example, for monitoring arrhythmias [24], nonmotor symptoms of Parkinson disease [25], and pressure ulcers [26]. However, most of these studies monitored a specific disease, and thus the platforms have limited applicability to studies of other diseases. Zens et al [18] developed a modular smartphone app that could be used in different medical studies without the need for advanced programming skills. Another example of an open-source framework is ResearchKit (Apple Inc). However, the initial studies revealed that technical programming skills, such as Object C or Swift, are needed to develop a functional app [27-29]. Another limitation is that ResearchKit only supports iOS devices. Other platforms, including ResearchStack and ResearchDroid, have been developed for use in research projects. ResearchStack is a functional software development kit with a framework comparable to that of ResearchKit for developing research apps for Android devices [30]. ResearchDroid is an Android library developed to automate survey forms and the information-building process [31]. Appbakery integrates ResearchKit and ResearchDroid, enabling researchers to create apps without requiring programming skills [32]. More recently, Google’s Open Data Kit has started allowing researchers to set up a study simply with a scalable app. Patient data can be exported to a comma-separated values file or viewed in the Google Cloud platform [33]. As examples of PC-based software, PsychoPy [34] and Labview (National Instruments) enable users to create individual software solutions with a graphical user interface in a process that does not require programming skills. The platform most similar to DHARMA was developed by Validic and can provide continuous access to personal health data obtained by over 350 in-home medical devices and wearables. Companies, such as Philips and IBM, also provide health platforms for remote monitoring. Although these commercially available platforms could have worked for the Premom research project, there were 3 main reasons why we chose to develop our own platform. First, because of the limited budget and the need for a vendor-independent research platform, we created our own solution that had the minimum number of components and required minimum development. Second, the platform needed to be flexible and customizable for use with new study (invalidated) thresholds. Third, we used integrated components and functions that could differ from the development roadmap for commercial platforms.

### Opportunities and Future Improvements

Although DHARMA provides exciting opportunities to improve remote monitoring services, it is not free from limitations. First, the data recorded by the medical devices are initially sent to the vendor’s dedicated database. This means that the vendor (eg, iHealth or Withings) also owns the patient’s data. An iHealth/Withings study profile without the patient’s personal data was created in the Premom study to deidentify the patients who were included in the follow-up program. This approach could be improved by creating a third-party app that connects directly to the medical devices; however, not all vendors allow direct access to their medical device via an open API. This process would bypass data transfer to an external company. A second limitation is the applicability of remote monitoring studies among technophobic individuals and people with limited cognition or ability to express consent, such as neonates, elderly, and sedated patients in an intensive care unit [35]. Related to this limitation, some people may not comply with manual entry of daily information, especially in long-term monitoring settings [36]. Automated *invisible* wearables, such as smartwatches or smart clothing, could help address noncompliance issues. Finally, it is difficult to keep pace with the rapidly evolving smartphone and wearable sensor technologies. Our platform was initially developed using PHP version 5.6.18, whereas the latest version is 7.4 at the time of writing. An advantage of DHARMA is its flexible architecture that enables rapid integration of new smartphone and wearable sensor technologies as they become available.

Currently, participants in DHARMA remote monitoring studies need to provide written informed consent to be enrolled in each study, as previously reported by Eysenbach et al [37]. Zens et al [18] described an alternative approach that uses an eligibility module to check the inclusion criteria and an integrated electronic informed consent component to obtain consent via a customized app. In the future, a similar component could be integrated into the DHARMA mobile app.

Another step to improve the platform will involve embracing the definitions of standard information models and information technology communication standards, such as Health Level 7 fast healthcare interoperability resources, together with clinical terminologies, such as systematized nomenclature of medicine—clinical terms, to ensure interoperability with hospital electronic medical record (EMR) systems [38]. Our platform can be seamlessly integrated into a patient’s daily life, but introducing it into a physician’s standard workflow may require integration with existing EMR systems.

### Conclusions

Smartphone health apps and medical devices collect large amounts of vendor-specific data. There are currently very few tools to collate and handle the data generated by multiple medical devices. We developed a component-based digital research platform to integrate the data in different formats from different sensors into a single integrated system. The platform performed well in a health care setting in real-time circumstances for the follow-up of pregnant women at risk of developing pre-eclampsia. The next stage in its development will involve integrating the platform with existing EMR systems to create a closed-loop information system.

Scientists or companies willing to contribute to this study are welcome to contact the authors.

## Abbreviations

AES: advanced encryption standard
API: app programming interface
DHARMA: digital health research platform for mobile health
ECG: electrocardiogram
EMR: electronic medical record
GDPR: general data protection regulation
PC: personal computer
